# APOA-5 Genetic Variant rs662799: Role in Lipid Changes and Insulin Resistance after a Mediterranean Diet in Caucasian Obese Subjects

**DOI:** 10.1155/2021/1257145

**Published:** 2021-08-12

**Authors:** Daniel de Luis, Olatz Izaola, David Primo

**Affiliations:** Endocrinology and Nutrition Research Center, School of Medicine, Department of Endocrinology and Nutrition, Hospital Clinico Universitario, University of Valladolid, Valladolid, Spain

## Abstract

**Background and Aims:**

This APOA5-1131C allele is related with a higher serum triglyceride levels and perhaps a different metabolic response to a dietary intervention. The aim of the present investigation was to evaluate SNP rs662799 in the *APOA5* gene and its associations with metabolic effects after a hypocaloric diet with Mediterranean pattern.

**Methods:**

A population of 363 Caucasian obese patients was enrolled. Anthropometric parameters and serum parameters (lipid profile, insulin, homeostasis model assessment (HOMA-IR), glucose, C reactive protein, adiponectin, resistin, and leptin levels) were measured, at basal time and after 3 months. All patients were genotyped in the rs662799 polymorphism.

**Results:**

The *APOA5* variant distribution was as follows: 89.3% (*n* = 324) (TT) were homozygous for the T allele, 10.5% (*n* = 38) (TC) were heterozygous, and 0.2% (*n* = 1) (CC) were homozygous for the C allele. Triglyceride levels were higher in patients with the C allele. After dietary intervention, BMI, weight, fat mass, waist circumference, systolic blood pressure, adiponectin, leptin, and adiponectin/leptin ratio improved significantly in both genotype groups TT and TC+CC. After dietary intervention, insulin levels (delta: −3.6 ± 0.8 UI/L vs. −1.5 ± 0.6 UI/L; *P* = 0.03), HOMA-IR (delta: −1.5 ± 0.4 units vs. −0.3 ± 0.2 units; *P* = 0.02), and triglyceride levels (delta: −19.3 ± 4.2 mg/dL vs. −3.2 ± 3.1 mg/dL; *P* = 0.02) decreased in non-C allele carriers.

**Conclusions:**

C allele carriers of rs662799 of the *APOA5* gene did not show an improvement in triglyceride, insulin, and HOMA-IR levels after a significant weight loss due to a hypocaloric diet with a Mediterranean pattern.

## 1. Introduction

Hypertriglyceridemia is a change in lipid profile commonly detected in obese subjects [1], and this metabolic alteration is considered an independent risk factor for atherosclerosis [2]. The role of apolipoprotein A5 (ApoA5) in the regulation of triglyceride metabolism has been demonstrated in several studies [3], this protein is composed of 336 amino acids, and it is contained in high-density lipoprotein (HDL) particles, and it is secreted from the liver [4]. This apolipoprotein is a well-known modulator of serum triglycerides with two mechanisms; it inhibits production of very low-density lipoprotein (VLDL) triglycerides, and it stimulates hydrolysis of VLDL triglycerides mediated by lipoprotein lipase [3, 4].

In addition, single-nucleotide polymorphisms (SNPs) in the *APOA5* gene have been related with triglyceride level metabolism and are considered important contributors to the development of different entities of metabolic syndrome [5–9]. Genetic variant rs662799 of the *APOA5* gene (-1131T>C) is considered to be functional-tag SNP [8, 9]. This genetic variant is associated with a higher serum triglyceride levels, and it contributes to increased risk of cardiovascular disease and the C allele impair ribosomal translation efficiency, thereby reducing the levels of APOA5 and reducing lipoprotein lipase activity [5–9]. Some experimental studies have evaluated whether changes in triglyceride levels depended on the combination of dietary intervention and this genetic variant. In a trial with patients with hypertriglyceridemia, TT homozygotes showed higher decrease in triglyceride levels [10]. After a low-fat/high-carbohydrate diet, females with the T allele showed a significant improvement in lipid profile [11]. Another study of high/low-fat diet showed that postprandial triglyceride elevation is affected by the T allele [12]. Finally, another investigation [13] has reported an interaction of the minor allele with high fat intake; these authors found highest triglyceride levels in subjects with the minor allele and high dietary fat intake.

As far as we know, there are no studies that have evaluated the relationship of this genetic variant with the influence of a hypocaloric Mediterranean diet. This is a research area with interest, since weight reduction improved lipid levels and this weight loss could be reach with a Mediterranean dietary pattern [14]. In addition, a diet with a Mediterranean pattern could have additional effects on lipid profile secondary to the foods with a direct action independently of weight change [15, 16].

Given its role in triglyceride levels and a lack of data in the literature, the current study was designed to evaluate SNP rs662799 in the *APOA5* gene and its associations with metabolic effects after a hypocaloric diet with a Mediterranean pattern.

## 2. Materials and Methods

### 2.1. Subjects

Study participants were recruited from the Health National Service during routine check-up of obesity of an urban area of the Northwest of Spain with a body mass index ≥ 30 kg/m^2^. 363 obese subjects agreed to participate in the study, and all participants provided written informed consent. The board approved the study, and the study was in accordance with the guidelines laid down in the Declaration of Helsinki. An ethical approval for genetic research was obtained, too. Inclusion criteria were the following: body mass index ≥ 30 kg/m^2^, no history of coronary events, no renal or hepatic disorders, normal thyroid hormone levels, alcohol intake less than 20 grams/day in women and less than 30 grams/day in men, and within the 9 months before the study were not receiving medications known to influence lipid levels (statins, fibrates, hormonal therapy, glucocorticoids, and anti-inflammatory drugs).

Data of these patients were recorded by an experienced investigator team at the beginning and after 3 months of dietary treatment. Classical anthropometric parameters such as weight, height, body mass index (BMI), and waist circumference were collected. Body fat mass was determined by electric impedanciometry. For biochemical assays and genotyping, 5 mL of venous blood was drawn and was aliquoted in ethylenediaminetetraacetic acid- (EDTA-) coated tubes after an 8-hour overnight fast. The following parameters were measured: lipid profile (total cholesterol, LDL-cholesterol, HDL-cholesterol, and triglycerides), C reactive protein (CRP), adipokine levels (leptin, total adiponectin, and resistin), fasting glucose, and insulin. Finally, homeostasis model assessment (HOMA-IR) and ratio adiponectin/leptin were calculated.

### 2.2. Anthropometric Parameters and Blood Pressure

Anthropometric parameters, like height (cm) and waist circumference (cm), were measured using a nonelastic measuring tape (Omrom, LA, CA). Body weight was recorded in the morning while the subjects were minimally unclothed and not wearing shoes, using digital scales (Omrom, LA, CA) and recorded to the nearest 50 g. Body mass index (BMI) was calculated as body weight (in kg) divided by height (in m^2^). Fat mass was measured by impedance with an accuracy of 5 g (EFG BIA 101 Anniversary, Akern, It). This equation was used: (0.756 Height^2^/Resistance) + (0.110 Body mass) + (0.107 Reactance)–5.463. Mean systolic and diastolic blood pressures were calculated by averaging four measurements (Omrom, LA, CA), after the subjects sat for 10 minutes.

### 2.3. Biochemical Procedures

The lipid profile formed by total cholesterol, HDL-cholesterol, and triglyceride levels was determined using the COBAS INTEGRA 400 analyser (Roche Diagnostic, Montreal, Canada). LDL-cholesterol was calculated using the Friedewald formula (LDL − cholesterol = total cholesterol − HDL cholesterol − triglycerides/5) [17]. Glucose levels were measured by an automated hexoquinase oxidase method (COBAS INTEGRA 400 analyser (Roche Diagnostic, Montreal, Canada). Insulin levels were determined by electrochemiluminescence assay (COBAS INTEGRA 400 analyser (Roche Diagnostic, Montreal, Canada). The homeostasis model assessment for insulin resistance (HOMA-IR) was calculated using these values (glucose × insulin/22.5) [18]. C reactive protein (CRP) was determined by immunoturbimetry (Roche Diagnostics GmbH, Mannheim, Germany).

Adipokine levels were measured by the enzyme-linked immunosorbent assay (ELISA). Leptin was determined with the commercial kit (Diagnostic Systems Laboratories, Inc., Texas, USA) (DSL1023100) [19]. Adiponectin was measured with other commercial kit (R&D systems, Inc., Mineapolis, USA) (DRP300) [20] and resistin with the kit (Biovendor Laboratory, Inc., Brno, Czech Republic) (RD191016100) [21].

### 2.4. Genotyping of *APOA5* Gene Polymorphism

Genomic DNA was extracted from 5 mL of whole blood using commercially available DNA isolation kit (Biorad, LA, CA). Primers were realized with the Sequenom Assay Design v4 (SEQUENOM, Inc., San Diego, California, CA). Genotyping for this SNP was performed by polymerase chain reaction real-time analysis. This polymerase chain reaction (PCR) was carried out with 25 ng of genomic DNA, 0.15–0.20 *μ*L each of oligonucleotide primer for rs662799 (primer forward: 5′-GAGCCCCAGGAACTGGAGCGAAAGT-3′ and reverse 5′-AGATTTGCCCCATGAGGAAAAGCTG-3′ in a 3.0 *μ*L final volume (Termociclador Lifetechnologies, LA, CA). DNA was denatured at 90°C for 5 min; this was followed by 45 cycles at 65°C for 15 s and annealing at 59°C for 40 s. The PCRs were run in a 2 *μ*L final volume containing 0.1 *μ*L of iPLEx Termination mix (Bio-Rad®, San Diego, CA) with hot start Taq DNA polymerase.

### 2.5. Nutritional Treatment

Participants were assigned to consume a calorie-restricted (daily 500-700 calories intake reduction) diet with a pattern of a Mediterranean diet for 3 months. All the subjects were given verbal and written instructions by a registered dietitian on completion of a 3-day (1 weekend and 2 week days) dietary record ant basal time and after 3 months of intervention. Records were analyzed with a computer-based data evaluation system (Dietsource ®, Ge, Swi). National composition food tables were used as reference [22]. The dietary intervention was a hypocaloric diet with a Mediterranean dietary pattern for 3 months. The balanced distribution of macronutrients in this diet was 50% of the calorie value from carbohydrates, 30% from lipids, and 20% from proteins. Percentage of fats was 55% from monounsaturated fats, 30% from saturated fats, and 15% from polyunsaturated fats. The diet was designed to include breakfast, lunch, dinner, one snack in the morning, and other snack in the afternoon. Food tables were used with a Mediterranean dietary pattern including (legumes, vegetables, poultry, whole grains, fish, fresh fruit, using olive oil, and limit unhealthy fats such as margarines, fatty meats, snacks, and industrial pastries) [22]. All participants had two individual sessions (60 minutes with diet sheets and example menu plans) with the dietitian at the start of the trail to explain the diet and solve doubts. The physical exercise allowed was an aerobic exercise at least 3 times per week (45-60 min each), and the patient with a self-reported questionnaire recorded it. To check participant's compliance during the whole study period, the dietitian interviewed them weekly by phone. They were interviewed to elucidate whether they were following the Mediterranean diet and the exercise activity.

### 2.6. Statistical Analysis

The sample size was calculated to detect differences over 15 mg/dL of triglyceride levels with 90% power and 5% significance. Statistical analyses were performed using SPSS for Windows, version 23.0 software package (SPSS Inc. Chicago, IL). All analyses were performed under a dominant genetic model with rs662799 C allele as the risk allele (TT vs. TC+CC). Descriptive statistics of all variable values are presented as mean ± standard deviation for continuous variables and as a number and percentage for categorical variables. Numerical variables with normal distribution were analyzed with two-tailed Student's *t*-test. Categorical variables were analyzed with the chi-square test, with Yates correction as necessary, and Fisher's test. The Bonferroni test was applied for multiple testing to reduce type I error in association analysis. The statistical analysis to evaluate the interaction between the gene and the dietary intervention was performed using ANCOVA (covariance analysis) adjusted by age, sex, and BMI modeling the dependent variable with the starting values. *P* values in Tables 1–3 are as follows: first *P*, significance of dietary intervention after 3 months in TT genotype; second *P*, significance between TT genotypes vs. TC+CC baseline values; third *P*, significance of dietary intervention after 3 months in TC+CC genotype; and fourth *P*, significance between TT genotypes vs TC+CC posttreatment values. The Hardy-Weinberg equilibrium was assessed with a statistical test (chi-square) to compare our expected and observed data.

## 3. Results

The *APOA5* variant distribution among the 363 obese subjects was as follows: 89.3% (*n* = 324) (TT) were homozygous for the T allele, 10.5% (*n* = 38) (TC) were heterozygous, and 0.2% (*n* = 1) (CC) were homozygous for the C allele. The allele frequency was T (0.94) and C (0.06). Considering that only one homozygous CC genotype was found, we pooled carriers of the less common allele C, as a dominant model (TC+CC). The variant of ApoA1 gene was in the Hardy-Weinberg equilibrium (*P* = 0.34)

The mean age was 49.8 ± 5.1 years (range: 25-65) and the mean body mass index (BMI) 36.2 ± 3.1 kg/m^2^ (range: 31.5-38.4). Gender distribution was 268 females (73.8%) and 95 males (26.2%). Mean values of age (TT; 49.9 ± 4.1 years vs. TC+CC; 49.5 ± 3.2 years: ns) as well as proportions of gender (TT 26.5% males vs. 73.5% females vs. TC+CC; 23.1% males vs. 76.9% females) were similar between both genotype groups. Patients reached the dietary recommendations as indicated in Materials and Methods, with a total caloric amount of 1555.1 ± 119.9 calories; the percentage of macronutrients was 50.8% from carbohydrates, 29.9% from lipids, and 19.3% from proteins. Percentage of fats was 55.4% from monounsaturated fats, 29.3% from saturated fats, and 14.3% from polyunsaturated fats, without statistical differences between genotype groups. Basal physical activity was similar in both genotype groups (TT vs. TC+CC) (113.1 ± 20.4 min/week vs. 118.1 ± 21.9 min/week; *P* = 0.52). Moreover, after 3 months of the study, this physical activity was similar (114.1 ± 19.8 min/week vs. 120.2 ± 31.8 min/week; *P* = 0.41), without statistical differences with basal exercise.

### 3.1. Anthropometric Results

Anthropometric parameters and blood pressure were not significantly associated with the APOA5 gene variant rs662799 (Table 1). After dietary intervention and in both genotype groups (TT vs. TC+CC), BMI (delta: −1.8 ± 0.2 kg/m^2^ vs. −1.9 ± 0.1 kg/m^2^; *P* = 0.56), weight (delta: −3.6 ± 2.1 kg vs. −3.7 ± 1.8 kg; *P* = 0.63), fat mass (delta: −2.4 ± 0.9 kg vs. −2.2 ± 1.0 kg; *P* = 0.53), waist circumference (delta: −4.1 ± 1.8 cm vs. −4.6 ± 1.9 cm; *P* = 0.48), and systolic blood pressure (delta: −5.1 ± 2.0 mmHg vs. −4.9 ± 2.1 mmHg; *P* = 0.38) decreased in a statistically significant way. This statistically significant improvement was similar in both groups.

### 3.2. Biochemical Parameters

Only triglyceride levels were higher in patients with the C allele delta in basal levels: −11.4 ± 3.9 mg/dL; *P* = 0.01 and delta in posttreatment levels −11.7 ± 3.9 mg/dL; *P* = 0.02. The remaining biochemical parameters were not significantly associated with the APOA5 gene variant rs662799 (Table 2).

The 3-month dietary intervention significantly decreased triglyceride levels, insulin levels, and HOMA-IR in non-C allele carriers. After dietary intervention (TT vs. TC+CC), insulin levels (delta: −3.6 ± 0.8 UI/L vs. −1.5 ± 0.6 UI/L; *P* = 0.03), HOMA-IR (delta: −1.5 ± 0.4 units vs. −0.3 ± 0.2 units; *P* = 0.02), and triglyceride levels (delta: −19.3 ± 4.2 mg/dL vs. −3.2 ± 3.1 mg/dL; *P* = 0.02) decreased in non-C allele carriers.

### 3.3. Adipokine Levels

Table 3 shows changes of serum adipokines and the calculated ratio adiponectin/leptin. After weight loss and in both genotypes (TT vs. TC+CC), serum adiponectin (delta: 18.2 ± 4.1 ng/dL vs. 15.2 ± 3.0 ng/dL; *P* = 0.31) increased in a significant way. In addition, patients with both genotypes showed a significant decrease on leptin levels (delta: −17.1 ± 5.2 ng/dL vs. −16.3 ± 4.9 ng/dL; *P* = 0.34). Serum resistin levels did not change after dietary intervention. Finally, the calculated adiponectin/leptin ratio increased in both genotypes (delta: 0.37 ± 0.1 vs.0.35 ± 0.2 ng/dL; *P* = 0.43).

No differences were detected among basal and postintervention adipokine levels between both genotypes.

## 4. Discussion

This genetic variant of *APOA5* gene (rs662799) was found to influence the 3-month dietary intervention targeting triglyceride and insulin levels in obese subjects. In non-C allele carriers, dietary intervention with a hypocaloric diet with a Mediterranean pattern produced significantly lower triglyceride, insulin, and HOMA-IR levels.

This result supports the idea that this genetic variant at *APOA5* locus may affect individual response to different treatments. For example, dislipemic subjects with the TT genotype have been reported to benefit more from statin treatment compared with C allele carriers [23]. Moreover, this SNP (rs662799) is associated with high levels of triglycerides [24], ischemic stroke [25], and coronary artery disease [26]. These data are consistent with the difference in triglyceride levels before and after treatment found in our study. This relationship can be explained because the C allele impairs ribosomal translation efficiency and thereby reduces the levels of APOA5 [27], and secondarily, this fact produced an impaired interaction with lipoprotein lipase activity and increased circulating triglyceride levels [28]. However, the low prevalence of the C allele has already been demonstrated in other studies [11, 12, 29–31], both in obese and normal weight patients in a range of 5-10%.

In the literature, some nutritional intervention studies have evaluated this SNP, although not using a diet with a Mediterranean pattern. In a previous study [11], Jang et al. demonstrated that dietary treatment decreased triglyceride levels in TT subjects with a 12-week dietary intervention without effect in C allele carriers. The intervention consisted of replacing 1/3 refined rice intake with legumes and increased vegetable intakes. This better response to dietary treatment is possibly due to changes in APOA5 concentration. The improvement in triglyceride levels in this study was 20%; in our study, the improvement was 15%; these changes in lipid concentration have been reported to be strongly associated to the subjects' initial lipid concentrations [10]. Another difference is that patients of this previous study [11] were hypertriglyceridemic patients, and the obese subjects of our study were nondislipemic patients. In other short-term intervention study [12], healthy young volunteers received a high-carbohydrate/low-fat diet for 6 days; proportions of carbohydrates, proteins, and fats were 7%, 15%, and 15%, respectively. C allele carriers had higher triglyceride levels after the diet than TT allele subjects. The C allele reduces the translational efficiency of APOA5, which in turn leads to the lower activity of lipoproteinlipase. Kim et al. [13] reported that nonobese healthy subjects than C carriers had delayed peak time of triglyceride and higher postprandial response of chylomicron after a high-fat meal compared to a low-fat meal. The high fat formula had 50.0% calories from fat and 34.6% from carbohydrate, and there were no significant differences in macronutrient intakes of the habitual diets. This impaired clearance of triglycerides in carriers of C allele may relate to a decrease in APOA5 function. In these subjects, the postprandial serum concentration of free fatty acids (FFA) and serum insulin concentration in C allele carriers were higher than noncarriers. In the postprandial state, the release of FFA into the circulation is determined by the intracellular hormone-sensitive lipase (HSL) and the extent to which lipoprotein lipase-derived fatty acids are not entrapped on adipose tissue and muscle [32]. Therefore, this lipid response observed in a postprandial state after a high-fat meal could be due to an impairment of the postprandial suppression of HSL by insulin in C allele carriers secondary to an insulin resistance state in these subjects. This situation could be implied in the lack of response of insulin and HOMA-IR in patients carrying the C allele in our study after dietary intervention. In a recent study in healthy volunteers [33], T allele carriers had higher postprandial lipidemia after an experimental breakfast of 75 g of fat, though the addition of glucose (25 g) to the test suppressed this difference. Perhaps secretion of insulin after glucose overload plays a role in these observations, because insulin action was shown to downregulate APOA5 expression and even to decrease ApoA-5 levels in plasma [34].

As far as we know, no study has been conducted evaluating dietary intervention with a Mediterranean pattern. In addition, our reported beneficial effects may be secondary to not only weight loss itself but also to the Mediterranean diet. The presence of low-calorie density foods and foods such as olive oil, fish, and lean meats with large amounts of fiber, polyphenols, monounsaturated fats, polyunsaturated, minerals, and vitamins can also explain the improvement in the profile lipid [14]. For example, Sanchez-Moreno et al. [29] found a genotype-dietary fat interaction with this genotype variant for obesity traits in a Mediterranean population in a cross-sectional study. In other cross-sectional study, Hubacek et al. [30] reported that triglyceride concentrations were higher in subjects with minor allele and the higher energy intake than non-C allele carriers. Lai et al. [35] reported that *n*-6 (but not *n*-3) polyunsaturated fatty acid intake increase fasting triglyceride concentrations in C allele carriers; therefore, the quality of the fat in the Mediterranean diet can also influence. Finally, calcium intake has been linked to this allele, too [36]. Carriers of the minor allele with low calcium intake (<500 mg/day) showed elevated triglyceride concentrations; it may be linked to cholesterol and bile acid intestinal reabsorption.

Some limitations should be considered when interpreting our findings. Firstly, dietary intake was based on self-reports obtained from patients by a registered dietitian with a potential bias. Second, the small size of C allele carriers and the results of genetic analyses should be interpreted with caution. Thirdly, the study has been designed in Caucasian obese subjects aged 20-60 years without dyslipemia, so the data are not generalizable to the entire population and different races. Fourthly, our study is a short-term dietary intervention, and this type of intervention might be more market than those observed in long-term dietary intervention as the latter could have an adaptative process which might result in a counterbalance of the initial response of the diet. Fifthly, the high prevalence of women in the study can make it difficult to generalize our results. Sixthly, the low prevalence of the T allele has already been shown in other studies [11, 12, 29–31]. Finally, circulating levels of ApoA5 have not been determined.

This study demonstrates that the minor C allele of APOA5 gene (rs662799) produces a worse response in triglyceride levels, insulin levels, and HOMA-IR after a hypocaloric diet with a Mediterranean pattern. Therefore, it would be necessary to detect early patients who are carriers of this allele in order to carry out a more powerful intervention. Further studies are necessary to evaluate the importance of this finding in the development of a more personalized medicine.

## Figures and Tables

**Table 1:** Anthropometric parameters and blood pressure (mean ± SD).

Parameters	TT (*n* = 324)		TC+CC (*n* = 39)		
	Basal	3 months	Basal	3 months	*P* values -Time TT -Basal genotype - Time TC+CC -3 months genotype
BMI	36.2 ± 1.1	34.4 ± 1.2^∗^	36.3 ± 1.2	34.6 ± 1.9^∗^	*P* = 0.02 *P* = 0.32 *P* = 0.01 *P* = 0.38
Weight (kg)	93.1 ± 1.2	89.5 ± 2.1^$^	92.1 ± 2.1	88.4 ± 2.0^$^	*P* = 0.03 *P* = 0.45 *P* = 0.02 *P* = 0.51
Fat mass (kg)	39.5 ± 1.2	37.1 ± 1.3^#^	40.4 ± 1.1	38.2 ± 1.7^#^	*P* = 0.03 *P* = 0.41 *P* = 0.02 *P* = 0.51
WC (cm)	109.2 ± 4.1	105.1 ± 3.1^&^	106.9 ± 3.4	102.2 ± 4.2^&^	*P* = 0.02 *P* = 0.31 *P* = 0.03 *P* = 0.41
SBP (mmHg)	129.7 ± 4.1	124.7 ± 2.9^∗∗^	129.1 ± 4.2	125.1 ± 3.1^∗∗^	*P* = 0.02 *P* = 0.33 *P* = 0.02 *P* = 0.43
DBP (mmHg)	83.3 ± 3.0	83.1 ± 4.1	81.9 ± 6.0	80.2 ± 5.0	*P* = 0.49 *P* = 0.61 *P* = 0.68 *P* = 0.61

BMI: body mass index; DBP: diastolic blood pressure; SBP: systolic blood pressure; WC: waist circumference; statistical differences *P* < 0.05, in each genotype group (^∗^BMI, ^$^weight, ^#^fat mass, ^&^WC, ^∗∗^SBP). First *P*: significance of dietary intervention after 3 months in TT genotype; second *P*: significance between TT genotypes vs. TC+CC baseline values; third *P*: significance of dietary intervention after 12 weeks in TC+CC genotype; fourth *P*: significance between TT genotypes vs. TC+CC posttreatment values.

**Table 2:** Biochemical parameters (mean ± SD).

Parameters	TT (*n* = 324)		TC+CC (*n* = 39)		
	Basal	3 months	Basal	3 months	*P* values -Time TT -Basal genotype -Time TC+CC -3 months genotype
Glucose (mg/dL)	101.8 ± 4.1	97.5 ± 7.0	97.7 ± 8.0	96.9 ± 7.1	*P* = 0.12 *P* = 0.51 *P* = 0.18 *P* = 0.40
Total cholesterol (mg/dL)	205.1 ± 4.7	194.9 ± 4.2^$^	209.2 ± 4.3	198.4 ± 4.2^$^	*P* = 0.02 *P* = 0.50 *P* = 0.03 *P* = 0.39
LDL-cholesterol (mg/dL)	127.9 ± 9.1	114.8 ± 5.2^#^	130.5 ± 6.0	109.2 ± 5.1^#^	*P* = 0.01 *P* = 0.49 *P* = 0.02 *P* = 0.35
HDL-cholesterol (mg/dL)	50.8 ± 1.6	52.2 ± 1.9	53.9 ± 2.1	51.7 ± 1.8	*P* = 0.13 *P* = 0.34 *P* = 0.59 *P* = 0.43
Triglycerides (mg/dL)	120.2 ± 8.0	100.9 ± 9.2^∗^	131.6 ± 6.2^+^	127.8 ± 5.1^+^	*P* = 0.02 *P* = 0.01 *P* = 0.31 *P* = 0.02
Insulin (mUI/L)	16.7 ± 1.1	13.1 ± 1.2^&^	13.3 ± 3.2	11.8 ± 2.9	*P* = 0.03 *P* = 0.41 *P* = 0.13 *P* = 0.49
HOMA-IR	4.6 ± 1.1	3.1 ± 1.0^∗∗^	3.0 ± 0.9	2.7 ± 1.1	*P* = 0.01 *P* = 0.38 *P* = 0.12 *P* = 0.49
CRP	4.4 ± 1.1	4.2 ± 1.0	4.5 ± 2.0	4.3 ± 2.1	*P* = 0.21 *P* = 0.31 *P* = 0.39 *P* = 0.40

HOMA-IR: homeostasis model assessment; CRP: C reactive protein. Statistical differences *P* < 0.05, in each genotype group (total cholesterol^$^, LDL-cholesterol^#^, insulin^&^, HOMA IR^∗∗^) (triglyceride between genotypes^+^). First *P*: significance of dietary intervention after 3 months in TT genotype; second *P*: significance between TT genotypes vs. TC+CC baseline values; third *P*: significance of dietary intervention after 12 weeks in TC+CC genotype; fourth *P*: significance between TT genotypes vs. TC+CC posttreatment values.

**Table 3:** Serum adipokine levels (mean ± SD).

Parameters	TT (*n* = 324)		TC+CC (*n* = 39)		*P* values -Time TT -Basal genotype -Time TC+CC -3 months genotype
	Basal	3 months	Basal	3 months	
Resistin (ng/dL)	3.9 ± 1.7	3.8 ± 2.1	4.0 ± 2.1	3.9 ± 1.1	*P* = 0.50 *P* = 0.61 *P* = 0.34 *P* = 0.41
Adiponectin (*μ*g/dL)	28.7 ± 4.0	46.9 ± 4.1^$^	27.7 ± 4.1	42.9 ± 4.2^$^	*P* = 0.01 *P* = 0.13 *P* = 0.02 *P* = 0.31
Leptin (ng/dL)	83.1 ± 9.6	66.2 ± 9.1^∗^	81.1 ± 5.1	63.8 ± 7.1^∗^	*P* = 0.01 *P* = 0.28 *P* = 0.02 *P* = 0.34
Ratio adiponectin/leptin	0.34 ± 0.2	0.71 ± 0.1^#^	0.33 ± 0.1	0.78 ± 0.3^#^	*P* = 0.02 *P* = 0.23 *P* = 0.03 *P* = 0.43

Statistical differences *P* < 0.05, in each genotype group (^$^adiponectin, ^∗^leptin, ^#^ratio adiponectin/leptin) (^+^adiponectin, ^++^adiponectin/leptin ratio between genotypes). First *P*: significance of dietary intervention after 3 months in TT genotype; second *P*: significance between TT genotypes vs. TC+CC baseline values; third *P*: significance of dietary intervention after 12 weeks in TC+CC genotype; fourth *P*: significance between TT genotypes vs. TC+CC posttreatment values.

## Data Availability

The authors can also make data available on request through an email to the corresponding author dadluis@yahoo.es Prof. Dr. Daniel de Luis. Some data are not freely available due to patient privacy.
